# Advancements in non-pharmaceutical interventions for Alzheimer’s disease management: an update review

**DOI:** 10.3389/fnagi.2026.1855269

**Published:** 2026-07-22

**Authors:** Jiali Chen, Ziyu Zhang

**Affiliations:** 1 Department of Global Medical Research Promotion, Shinshu University Graduate School of Medicine, Matsumoto, Japan; 2 Department of Geriatrics II, Aerospace Center Hospital, Beijing, China

**Keywords:** Alzheimer’s disease, non-pharmaceutical interventions, physical activity, dietary therapies, cognitive stimulation therapy

## Abstract

Alzheimer’s disease (AD) is a chronic, progressive neurodegenerative condition that is characterized by an increasing incidence rate due to global population aging, resulting in a significant social and economic burden. Current pharmacological interventions offer only limited relief and are unable to halt neuronal loss and cognitive decline. Therefore, safe and effective non-pharmacological interventions (NPIs) are needed for the management of AD. This review systematically synthesizes literature from the PubMed and Web of Science databases (2020–2025) to evaluate the comparative efficacy of physical activity, dietary interventions, and cognitive stimulation therapy (CST), aiming to establish an evidence-based framework for optimized clinical implementation. Specifically, physical activity (e.g. aerobic exercise and strength training) has been shown to improve cognitive and physical function. Dietary interventions (e.g. the Mediterranean diet, Dietary Approaches to Stop Hypertension and The Mediterranean-DASH diet intervention for neurodegenerative delay diet) have been demonstrated to delay cognitive decline by modulating inflammation, but evidence regarding supplements is weak. Furthermore, CST has been evidenced to enhance patients’ cognitive abilities and quality of life. The present review not only summarizes existing literature on the subject, but also explores future research directions, including mechanisms and personalized approaches of these interventions to improve the prognosis of AD patients.

## 1 Introduction

Dementia is a syndrome that can be caused by a number of diseases and damage to nerve cells, and affects brain function over time, leading to a decline in cognitive ability. Alzheimer’s disease (AD) is the most common type of dementia (accounting for 60%–70% of all cases), serves as a hallmark neurodegenerative disorder; it is characterized by the insidious onset of cognitive impairment–specifically affecting memory, judgment, and problem-solving (Alzheimer’s Disease International, 2024). It is characterized by the extracellular accumulation of plaques containing β-amyloid (Aβ) and the intracellular formation of tangles of tau protein-containing neuroproteins fibers (Zhang et al., 2024). Pathologically, it initially manifests as synaptic dysfunction, progressing to neurodegeneration and neuronal cell death in later stages (Abdulkhaliq et al., 2026). The most recent data indicate that the epidemiology of AD and general dementia are closely related (Scheltens et al., 2021). The latest data from the World Health Organization (WHO) shows that, approximately 55 million people are currently living with dementia worldwide, with almost 10 million new cases arising annually (World Health Organization, 2022). Given its prevalence and aging global population, it is becoming a global health problem, with individual and societal implications. Furthermore, AD patients primarily present as progressive cognitive impairment and behavioral deficits, negatively impacting the health and quality of life of older individuals. These symptoms also place a financial burden on families and society (Alzheimer’s Association, 2026; Ren et al., 2022).

Currently, drug interventions for AD focus primarily on symptom management (Mertas and Bosgelmez, 2025). Consequently, there is an imperative for the development of novel, effective and safe non-pharmaceutical interventions (NPIs) that can intervene and treat AD. NPIs have been demonstrated to be both cost-effective and devoid of adverse reactions, whilst concomitantly exerting a favorable influence on the quality of life of patients afflicted with cognitive impairment and their caregivers (Buccellato et al., 2023). The objective of this study is to proposes an integrated therapeutic framework to elucidate the synergistic effects of physical activity, dietary therapy, and CST, thereby providing a structured, evidence-based approach to optimize outcomes in neurodegenerative management.

A systematic review and meta-analysis showed that multi-domain lifestyle interventions (combining physical exercise and cognitive training) can effectively modulate the overall cognitive abilities of older adults with normal cognitive function (Mendes et al., 2025). In addition, recent scoping evidence underscores the superior efficacy of multidomain interventions over single-modality approaches, providing a foundational basis for the shift toward integrative, patient-centered AD management (Roach et al., 2025). A systematic review (Bereczki et al., 2025) synthesizing evidence on the integration of lifestyle and pharmacological/nutraceutical interventions, provides a compelling framework for precision prevention across the AD continuum, highlighting the synergistic potential of combining multi-target strategies. The complex pathophysiological mechanisms and heterogeneity of AD have long limited the efficacy of clinical interventions. While previous reviews have largely focused on specific pathological pathways or traditional drug therapies, there has been little systematic analysis of existing non-pharmacological approaches. By systematically integrating existing clinical evidence on non-pharmacological interventions, this article provides clinicians with actionable intervention strategies and offers prospective theoretical support for transforming AD research toward “prevention and personalized management.”

## 2 Overview of present therapeutic interventions

Presently, pharmaceutical interventions are the treatment modality for patients with AD worldwide. The following primary drug therapies are currently employed in clinical practice: The following substances have been identified as effective treatment options for AD: (1) Cholinesterase inhibitors, including donepezil, galantamine, rivastigmine and memantine; (2) Anti-β-amyloid monoclonal antibodies, such as donanemab and lecanemab (Fox et al., 2025; Frisoni et al., 2025; Mintun et al., 2021; Van Dyck et al., 2023). Despite the current availability of pharmacological options, their clinical efficacy remains limited. These medications primarily focus on managing symptoms rather than addressing the underlying disease-modifying mechanisms. Consequently, they fail to halt progressive neuronal loss, brain atrophy or the decline of cognitive function. Furthermore, the therapeutic landscape is complicated by the significant risks associated with polypharmacy, which is a critical concern in the geriatric population and often exacerbates adverse outcomes. The lack of effective disease-modifying treatments highlights the urgent public health challenge of managing AD and related dementias. The utilization of polypharmacy has been demonstrated to enhance the prevalence of unfavorable reactions and adverse effects, concurrently diminishing medication adherence (Kwon et al., 2024).

Consequently, there is an imperative for the development of effective NPIs to alleviate symptoms in AD patients and reduce the significant social burden. In consideration of the characteristics of pharmacological treatment, the application of NPIs in the intervention and treatment of AD has garnered widespread attention.

**FIGURE 1:**
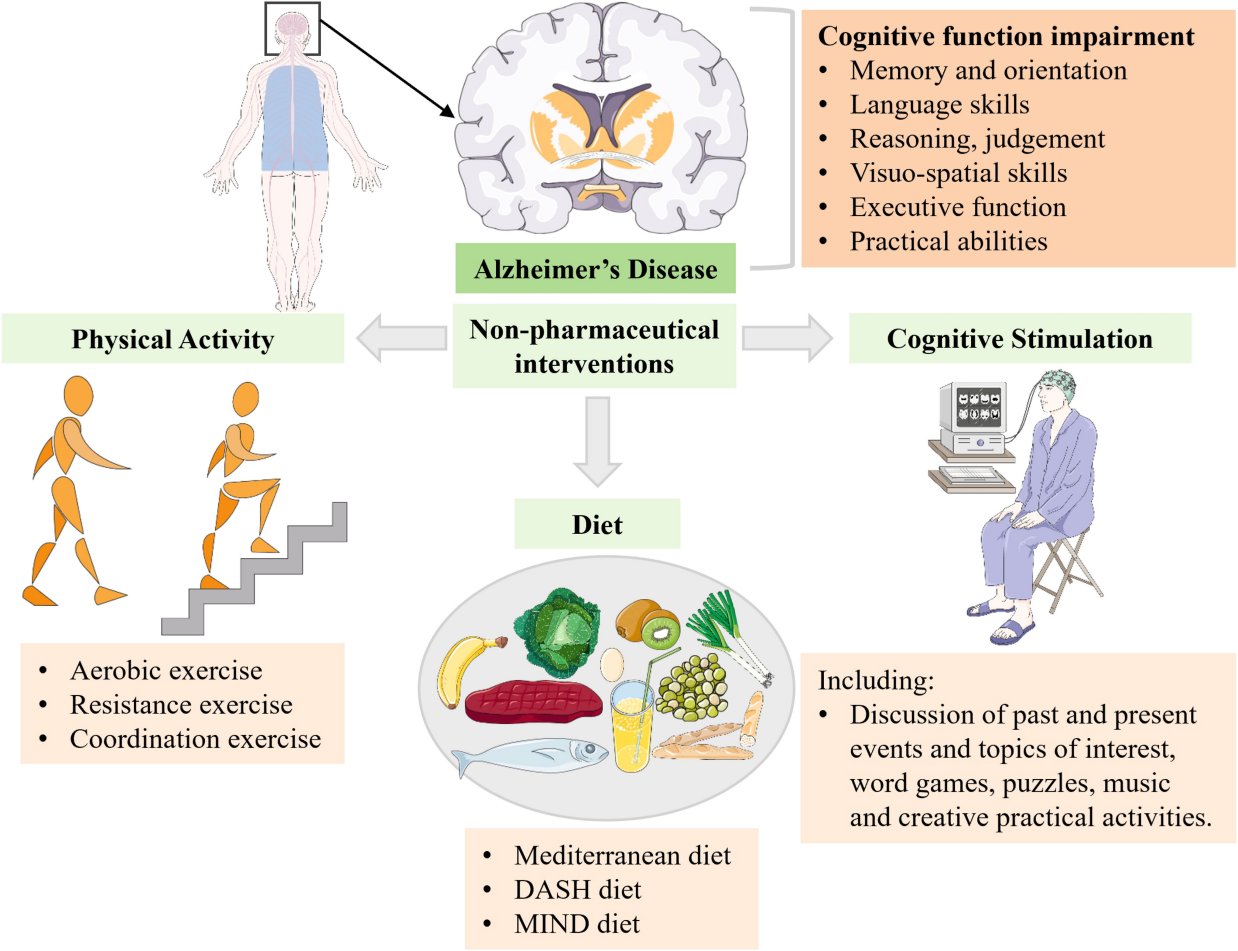
Clinical manifestation of AD and NPIs (physical activity and diet and cognitive stimulation).

Non-pharmacological interventions refer to any medical interventions that are not based on pharmacotherapy. They can be used to prevent or treat diseases and other health conditions, or to improve public health. NPIs, including cognitive behavioral therapy and environmental modifications, aim to help patients develop effective coping mechanisms, reduce distress and enhance their overall quality of life (He et al., 2023). Several studies have shown that NPIs play an important role in the treatment of AD and can be effective in improving patients’ cognitive function, emotional state and overall health (Jia et al., 2021; Yu et al., 2019). Beyond their established roles in symptom management and quality-of-life enhancement, emerging evidence suggests that non-pharmacological interventions (NPIs) exert meaningful biological effects on the AD brain (Reuben et al., 2024). At the cellular level, NPIs promote neuroplasticity by increasing synaptic density and upregulating neurotrophic factors (such as BDNF) (Kandola et al., 2019; Marosi and Mattson, 2014; Oyovwi et al., 2025; Sanada et al., 2016). Physiologically, NPIs may foster a neuroprotective milieu by attenuating chronic neuroinflammation (such as NLRP3 inflammasome activation), and by improving cerebral glucose metabolism, thereby counteracting key drivers of neuronal atrophy (Greco et al., 2016; Hu et al., 2024; Park et al., 2025; Zhao et al., 2025). Collectively, these findings suggest that NPIs should be regarded not merely as adjunctive supportive measures, but as strategic, disease-modifying approaches that enhance neural resilience and may slow the underlying neurodegenerative process in AD. Through literature review, we found that the following NPIs may have beneficial effects on patients with AD (Figure 1).

## 3 Physical activity interventions

Physical activity is defined as planned, structured, and repetitive movement intended to enhance or preserve one or more components of physical fitness. It is comprised of several components, including type, dose, and duration. Contemporary evidence concerning the impact of exercise training is considered for a range of exercise types, including aerobic, strength, flexibility, and balance training (Chodzko-Zajko et al., 2009). Physical activity or “exercise” is a significant modifiable factor in the pathophysiological development of AD (Alty et al., 2020; Valenzuela et al., 2020), with low physical activity being significantly associated with a high AD risk (Zhang et al., 2021). The results of a meta-analysis indicate that Physical activity can reduce the risk of developing AD. Furthermore, physical activity has been demonstrated to enhance cognitive function and physical performance in individuals with AD (Demurtas et al., 2020; López-Ortiz et al., 2021, 2023). A systematic review demonstrated that physical activity intervention may enhance subjective sleep quality in AD’s patients, with greater efficacy observed in patients with severe underlying sleep disturbances or those who engage in ≥1 h of exercise (Tan et al., 2026).

Physical activity is a promising NPIs for AD, as it induces multi-systemic physiological adaptations that extend beyond cardiovascular health. Mechanistically, regular exercise promotes the secretion of neurotrophic factors, particularly BDNF, which supports synaptic plasticity and hippocampal neurogenesis–processes often impaired in the early stages of AD (Erickson et al., 2011; Lourenco et al., 2019; Szuhany et al., 2015). Furthermore, physical activity exerts neuroprotective effects by mitigating chronic neuroinflammation and inhibiting the overactivation of microglia (De Almeida et al., 2022). Emerging evidence also highlights its role in brain waste clearance; by improving cerebral hemodynamics, physical activity facilitates the influx of cerebrospinal fluid within the glymphatic system and enhances drainage through meningeal lymphatic vessels (mLVs), thereby assisting in the removal of neurotoxic proteins (Rehman et al., 2024; Yoo et al., 2025).

Physical activity interventions for AD can be delivered through a specific exercise programmer of moderate to high intensity (usually aerobic or resistance exercise) or lower intensity, depending on the patient’s own exercise habits (Broadhouse et al., 2020); interventions are varied and can be single-task, dual-task or multi-task training (Menengi et al., 2022). To summaries, physical activity interventions represent an approach to the maintenance and enhancement of physical and functional abilities in individuals diagnosed with AD (Valenzuela et al., 2020). Furthermore, physical activity has been shown to enhance cognitive function and mitigate the risk of falls in individuals with AD (Cezar et al., 2021; Yang et al., 2025). Table 1 summaries the physical activity intervention clinical trials for people with AD that published between 2020 and 2025 (David et al., 2025; Gaitán et al., 2021; Kim et al., 2024; Marston et al., 2024; Yu et al., 2021).

**TABLE 1:** Physical exercise interventions.

Date: 2020–2025							
References	Country	Groups		Type of exercise	Duration	Intervention	Primary outcomes
		Intervention group	Control group				
Zhang et al., 2021	China	*n* = 64	*n* = 32	Aerobic exercise	6-month	Moderate-intensity cycling for 20–50 min, 3 times/week; Stretching group: light-intensity.	(−) ADAS-Cog
López-Ortiz et al., 2023	United States	*n* = 11	*n* = 12	Aerobic exercise	26-week	UPA group: lifestyle education; EPA group: moderate-to-vigorous intensity aerobic exercise (3 times/week).	↑Plasma CTSB; ↓BNDF; (−) klotho levels
Demurtas et al., 2020	Germany	*n* = 22	*n* = 19	Aerobic, resistance, coordination exercise	6-month	Intervention group: moderate-to-vigorous intensity home-based training for at least 30 min/week + stretching and toning exercises for at least 15 min, twice/week; Control group undergoing a psychoeducational program.	↑VO2max; ↑MoCA; ↑postural stability score
López-Ortiz et al., 2021	Australia	*n* = 62	*n* = 29	Aerobic exercise	6-month	High-intensity and moderate-intensity exercise group: undertook 50-min exercise sessions twice a week, 6-month; control group: no exercise.	↑CTSD/APBB3; ↓CDK5R1/ ADAM17/ MAPK1/ MAPK/ PSENEN/ CASP3/ CAPNS2
Tan et al., 2026	Korea	*n* = 36	*n* = 19	Aerobic, resistance exercise	12-week	AG: aged 60–69 walk 35000–50000 steps/week; aged 70–79 walk 30000–45000 steps/week; aged 80–89 walk 20000–35000 steps/week; RAG: resistance exercise + walking (2 times/week; CG: routine daily activities + light-intensity stretching (1 h, 2 times/week).	↓AD7c-NTP

ADAS-Cog, Alzheimer’s Disease Assessment Scale-Cognition; UPA, usual physical activity; EPA, enhanced physical activity; CTSB, Cathepsin B; BDNF, brain-derived neurotrophic factor; MoCA, Montreal Cognitive Assessment; CTSD, Cathepsin D; APBB3, Amyloid beta precursor protein binding family B member 3; CDK5R1, cyclin dependent kinase 5 regulatory subunit 1; ADAM17, A disintegrin and metalloprotease 17; MAPK1, mitogen-activated protein kinase 1; PSENEN, presenilin enhancer, gamma-secretase subunit; CASP3, Caspase 3; CAPNS2, calpain small subunit 2; AG, aerobic exercise group; RAG, resistance/aerobic exercise group; CG, active control group; AD7c-NTP, Alzheimer-associated neuronal thread protein. ^↑^ Indicates an increase or upregulation of the measured parameter. ^↓^ Indicates a decrease or downregulation of the measured parameter.

Research has shown that, resistance training, or aerobic exercise lasting more than 6 months, 4–5 times/week, and lasting no more than 30 min per session, may be the most effective way to improve activities of daily living (ADLs) in patients with AD (Xiao et al., 2024). Additionally, a systematic review and meta-analysis of existing studies on exercise therapy revealed that aerobic exercise lasting more than 16 weeks and performed ≥3/week for 30–50 min per session can improve MMSE scores, ADAS-Cog scores, and quality of life in patients with AD (Gaitán et al., 2021). However, it is important to note that high-intensity exercise, particularly when sustained over a 3-months intervention period, has been reported to induce a stronger pro-inflammatory state than moderate-intensity exercise (Khakroo Abkenar et al., 2019).

Therefore, there are several considerations when providing physical activity interventions for AD patients: (1) Development of a personalized exercise programmer based on each patient’s physical condition, interests and abilities. (2) Regular assessment and adjustment of the exercise programmer according to the patient’s feedback and progress. (3) Safe exercise environment: Ensuring the safety of the exercise venue to avoid falls and other accidents. (4) Provision of emotional support: The involvement of family carers is encouraged to enhance patients’ enjoyment and motivation during exercise. (5) Exercise records: Patients, family carers or healthcare workers should be advised to document the frequency and emotional aspects of exercise. (6) Effective feedback mechanisms: Regular communication with the healthcare team is essential to evaluate the effectiveness of the exercise programmer and make necessary adjustments.

## 4 Dietary therapies

Dietary therapy, categorized as a form of NPIS, involves the modification of dietary habits and food intake with the objective of adjusting a specific indicator of physical and mental health (Ahn et al., 2024; Kurowska et al., 2023). It has the capacity to change the dietary habits of patients in their daily lives, thereby slowing down the progression of the disease (Levak et al., 2024). The Dementia Prevention Nutrition Working Group recently found that dietary therapy may directly or indirectly influence cognitive performance in AD through a complex interplay of behavioral, genetic, systemic and brain factors (Jiang et al., 2023).

It is evident that dietary interventions are more than simply a means of providing basic nutrients; they also actively regulate key molecular pathways. Recent research highlights the “brain-gut-microbiota axis” as a major regulator of neuro-inflammation, it is crucial for maintaining brain homeostasis and preventing disorders (Kowalski and Mulak, 2019; Liu et al., 2020; Schächtle and Rosshart, 2021). At the cellular level, specific nutrients have been shown to restore insulin sensitivity through the PI3K/Akt pathway. The process under discussion has been demonstrated to inhibit GSK-3β activity, thus helping to reduce the harmful buildup of tau proteins (Ozpak and Bağca, 2024). Furthermore, precision nutrition has been demonstrated to enhance mitochondrial function and overall metabolic health (Bredesen et al., 2023). By targeting these specific pathways, dietary interventions have been shown to help protect against the synaptic damage that is characteristic of AD.

Diet has been identified as a modifiable lifestyle factor with the ability to influence the maintenance of cognitive health (Arora et al., 2023; Charisis et al., 2025; Ellouze et al., 2023). Since nutrients play a role in maintaining cognitive function, neuronal function depends on an adequate supply of nutrients. There is a close relationship between many nutrients and the onset and development of degenerative diseases of the central nervous system (Ayten and Bilici, 2024; Nie et al., 2024). Therefore, dietary therapy could be an effective way to prevent the progression of AD. Table 2 summaries the dietary therapy intervention trials for people with AD that published between 2020 and 2025 (Arellanes et al., 2020; Chen et al., 2021; Choi et al., 2022; Fei et al., 2023; Fernando et al., 2023; Foroumandi et al., 2023; Fortier et al., 2021; Jung et al., 2021; Lin et al., 2022; Nolan et al., 2022; Phillips et al., 2021; Sala-Vila et al., 2020; Tamtaji et al., 2023; Thota et al., 2020; Tofiq et al., 2021; Xiao et al., 2020).

**TABLE 2:** Dietary therapy interventions.

Date: 2020–2025						
References	Country	Groups		Intervention	Duration	Primary outcomes
		Intervention group	Control group			
Gaitán et al., 2021	Barcelona, Spain; Loma Linda, CA	*n* = 336	*n* = 321	The walnut group: 15% of daily energy (30–60 g/d) intake as walnuts; control diet (abstention from walnuts).	2-year	(−) Global cognitive composite score
David et al., 2025	Australia	*n* = 14	*n* = 15	Curcumin (2 × 500 mg curcumin tablets [Meriva^®^] providing 180 mg of curcumin per day); placebo group (2 × placebo tablets matching for curcumin).	12-week	↓ GSK-3β; IAPP; insulin resistance
Marston et al., 2024	Japan	*n* = 40	*n* = 39	Probiotic group: receive once daily either probiotic (*Bifidobacterium breve* A1, 2 × 1010 Colony Forming Unit); Placebo group capsules were composed of maize starch only.	16-week	↑RBANS total score, JMCIS score
Kim et al., 2024	United States	*n* = 18	*n* = 15	DHA treatment: 538 mg DHA soft-gel capsules per day; Placebo: identically-appearing capsules containing corn/soy oil manufactured.	6-month	↑CSF DHA levels, CSF EPA levels, (−) hippocampal volume and cognitive scores
Xiao et al., 2024	Canada	*n* = 39	*n* = 43	The kMCT drink treatment: ketogenic medium chain triglyceride [kMCT]; 15 g twice/day; Placebo: high-oleic acid sunflower oil as a calorie-equivalent non-ketogenic vegetable oil.	6-month	↑ Neurocognitive battery
Khakroo Abkenar et al., 2019	Australia	*n* = 13	*n* = 13	Intervention diet: modified ketogenic diet; Control diet: usual diet	12-week	↑ ADCS-ADL and QOL-AD
Ahn et al., 2024	China	*n* = 51	*n* = 50	Intervention group: three tablets of 400 pg of folic acid (1.2 mg/d) and two tablets of 25 pg of vitamin B12 (50 pg/d); Placebo group received three starch tablets similar to folic acid tablets and two starch tablets similar to the vitamin B12 tablets.	6-month	↑ MoCA total scores, naming scores, orientation scores, ADAS-Cog domain score of attention
Kurowska et al., 2023	Sweden	*n* = 18	*n* = 15	Intervention group: four 1 g capsules daily, each containing 0.43 g DHA and 0.15 g EPA, making a total of 2.3 g of n-3 FAs per day; Placebo group: four isocaloric 1 g capsules daily, containing 1 g corn oil (including 0.6 g linoleic acid, an omega-6 fatty acid).	6-month	(−) MMSE; ↑ YKL-40 and NfL
Levak et al., 2024	Korean	*n* = 35	*n* = 35	The SOCE group took a total of 3 g (Including 1.5 g/day as SOCE) per day, orally three times a day (before breakfast, lunch, and dinner); The placebo group took 3 g (0 g/day as SOCE) orally by consuming one tablet before breakfast, lunch, and dinner every day.	12-week	↓ Amyloid-β (1–40) and amyloid-β (1–42) levels
Jiang et al., 2023	China	*n* = 123	*n* = 40	EPA group: 1.6 gm EPA daily; DHA group: 0.7 gm DHA daily; EPA + DHA group: 0.8 gm EPA and 0.35 gm DHA daily; Placebo group: soybean oil.	24-month	EPA: ↓CCL4 level, constructional praxis, spoken language ability scores
Schächtle and Rosshart, 2021	Ireland	*n* = 50	*n* = 27	Intervention group: 1 g fish oil (of 500 mg DHA, 150 mg EPA), 22 mg carotenoids (10 mg lutein, 10 mg meso-zeaxanthin, 2 mg zeaxanthin), and 15 mg vitamin E daily; Placebo group: sunflower oil.	12-month	↑ Skin carotenoid concentrations
Kowalski and Mulak, 2019	Korea	*n* = 40	*n* = 40	SM70EE group: 1 g/day of SM70EE, 3 times/day for 12 weeks; Placebo group: caraway extract (1 g/day).	12-week	(−) K-MMSE score; ↑ CNT
Liu et al., 2020	Iran	*n* = 38	*n* = 40	Intervention group: 5 cc orally of fenugreek seed extract (equivalent to 500 mg of dry extract); Placebo group: simulated plain water with a taste and color similar to that of fenugreek seed extract.	4-month	↑ Serum levels of TAC; ↓ MDA status
Ozpak and Bağca, 2024	Iran	*n* = 30	*n* = 30	Patients were randomly assigned to receive either 500 mg/day spirulina or a Placebo twice a day.	12 weeks	↑ MMSE score and insulin sensitivity; ↓ hs-CRP, fasting glucose, insulin and insulin resistance
Bredesen et al., 2023	China	*n* = 21	*n* = 21	Probiotic group consumed 2 g probiotics daily; Control group consumed a 2 g starch capsule placebo daily.	12-week	↑ Cognitive function and sleep quality
Charisis et al., 2025	Sri Lanka	*n* = 43	*n* = 41	The intervention group: 30 mL/day of VCO orally; Control group: similar amount of canola oil.	24-week	(−) Cognitive scores, lipid profile and HbA1 C levels; ↑ MMSE score in APOE ϵ4 carriers

GSK-3β, Glycogen synthase kinase-3β; IAPP, Islet amyloid polypeptide; RBANS, Repeatable Battery for the Assessment of Neuropsychological Status; JMCIS, Japanese version of the MCI Screen tests; CSF, Cerebrospinal fluid; DHA, Docosahexaenoic acid; EPA, Eicosatetraenoic acid; ADCS-ADL, AD Cooperative Study - Activities of Daily Living inventory; QOL-AD, Quality of Life in AD; MoCA, Montreal Cognitive Assessment; ADAS-cog, Alzheimer’s Disease Assessment Scale-Cognitive subscale; MMSE, Mini-Mental State Examination; YKL-40, Chitinase-3-like protein 1; NfL, Neurofilament light; SOCE, Sesame oil cake extract; CCL4, C-C motif ligands 4; SM70EE, Spirulina maxima 70% ethanol extract; K-MMSE, Korean mini-mental status examination; CNT, Computerized neurocognitive test; TAC, Total antioxidant capacity; MDA, Malondialdehyde; hs-CRP, high-sensitivity C-reactive protein; VCO, Virgin coconut oil; HbA1 C, Glycosylated Hemoglobin, Type A1C; APOE, apolipoprotein E ϵ4. ^↑^ Indicates an increase or upregulation of the measured parameter. ^↓^ Indicates a decrease or downregulation of the measured parameter.

Growing evidence suggests that dietary patterns have significant effects on cognitive function and risk of AD (Chu et al., 2022). At present, several dietary patterns have been shown to have a positive effect on AD patients (Morris et al., 2015a, b). (1) Mediterranean diet: The specific food components included in the Mediterranean diet are shown in Table 3. Currently, the Mediterranean diet has been employed extensively in the intervention and treatment of neurological diseases, such as Parkinson’s Disease and AD (Knight et al., 2022; Petersson and Philippou, 2016). Moreover, it has been demonstrated to decelerate the progression of cognitive decline and mitigate the likelihood of developing mild cognitive impairment and AD (Zupo et al., 2025). (2) Dietary Approaches to Stop Hypertension (DASH) diet: Compared with the Mediterranean diet, the DASH diet also emphasizes a high intake of plant-based foods. However, unlike the Mediterranean diet, it emphasizes low sodium intake and reduced consumption of sugary beverages and red meat. It also does not recommend alcohol consumption (Table 3). The DASH diet has been demonstrated to have a positive effect on cognitive function (Mancilla and Rojas, 1990; Tangney et al., 2014). Moreover, it has the potential to mitigate the risk of developing AD (Amini et al., 2020). (3) The Mediterranean-DASH diet intervention for neurodegenerative delay (MIND) diet: This dietary plan integrates the benefits of the Mediterranean diet and the DASH Diet, with a particular emphasis on the consumption of dietary components that have been shown to have neuroprotective effects, such as berries and leafy green vegetables (Table 3). The MIND diet has been shown to slow cognitive decline in older adults (Agarwal et al., 2024). It can also improve cognitive function and lower the risk of dementia (Kheirouri and Alizadeh, 2022; Van Soest et al., 2024).

**TABLE 3:** Summary of features of the Mediterranean diet, DASH and MIND diets.

Dietary patterns	High consumption	Moderate consumption	Low consumption
Mediterranean diet	Non-refined cereals and products, fresh fruit, vegetables, olive oil, dairy products, wine in moderation.	Fish and seafood potatoes, olives, pulses, nuts, eggs, sweets.	Red meat, dairy products (mostly in the form of cheese and yogurts).
DASH diet	Whole grains, fruit, vegetables, fat-free or low-fat dairy products, nuts, seeds.	Fish, poultry.	Salt, saturated fat, sugar-sweetened beverages and sweets, red and processed meats.
MIND diet	Whole grains, fish, fresh fruit, green leafy, other vegetables, olive oil, beans, nuts, poultry.	Red meat and products, alcohol/wine.	Pastries and sweets, cheese, butter/margarine, fast fried foods.

Taken collectively, the available evidence suggests that the following dietary ingredients should be considered for implementation in dietary interventions targeting AD patients: fresh fruit and vegetables, whole grains (e.g., oats, brown rice), good fats (e.g., olive oil, fish oil) and high-protein foods (e.g., fish, poultry, beans). It is recommended that limits or avoidance of the following be considered: refined sugars and processed foods, saturated and trans fats, high-salt foods. By implementing sensible dietary therapies, we hope that the progression of AD can be slowed, cognitive function can be protected, and the risk of developing the disease can be reduced.

## 5 Cognitive stimulation therapy (CST)

Cognitive training constitutes a non-pharmacological form of treatment that focuses on guided practice of tasks designed to target specific cognitive functions. As a specialized subset of cognitive training, cognitive stimulation therapy (CST) functions as an evidence-based, structured psychosocial intervention specifically designed for people with dementia (National Collaborating Centre for Mental Health (UK), 2007; Orrell et al., 2017). It consists of 14 group sessions designed to stimulate different cognitive brain functions (Spector et al., 2010). Numerous studies have demonstrated its efficacy in promoting language function in individuals diagnosed with dementia (Spector et al., 2010). In addition, CST has been shown to have a significant impact on the cognitive function and quality of life of people with mild-to-moderate dementia, leading to a reduction in depressive symptoms (Saragih et al., 2022; Spector et al., 2003; Woods et al., 2012). Due to space limitations, this article cannot provide a comprehensive discussion of existing CST therapies. However, a literature search revealed a systematic review showing that CST can enhance language abilities and short-term memory, thus supporting personalized rehabilitation for patients with early cognitive impairment (Liang et al., 2025). Furthermore, a systematic review and Bayesian meta-analysis suggest that showing that cognitive training, the foundational framework of CST, is more effective in improving overall cognitive abilities in patients with AD and other dementias (Giaquinto et al., 2025). Table 4 summaries the CST intervention clinical trials for AD patients that published between 2020 and 2025 (Alvares-Pereira et al., 2021; Bertrand et al., 2023; Carbone et al., 2021; Gibbor et al., 2021; Lok et al., 2020; Marinho et al., 2021; Mori et al., 2024; Rai et al., 2021).

**TABLE 4:** Cognitive stimulation therapy interventions.

Date: 2020-2025						
References	Country	Groups		Intervention	Duration	Primary outcomes
		Intervention group	Control group			
Chu et al., 2022	Turkey	*n* = 30	*n* = 30	The experimental group: treated using RAM-based CST; Control group: routine treatment (monthly polyclinic monitoring). For 7 weeks (2 days a week).	7 weeks	↑QOL-AD, ↑CAPS&SMMSE
Morris et al., 2015a	UK	*n* = 16	*n* = 13	iCST: 14-session structured program, 45-min; TAU: treatment as usual (three 30-min sessions a week).	7 weeks	↓ADAS-Cog; (−) SMMSE
Morris et al., 2015b	Brazil	*n* = 23	*n* = 24	CST group: 4 sessions of CST + treatment as usual; TAU: treatment as usual. Twice a week, over 7 weeks.	7 weeks	(−) ADAS-Cog, ↓CSDD, (−) ADCS
Knight et al., 2022	Portugal	*n* = 55	*n* = 50	CST: CST training; TAU: treatment as usual. Twice a week for 7 weeks, with each session lasting 45–60 min.	7 weeks	↑ADAS-Cog, HCS, CDR, CAPE-BRS, (−) RAID, CSDD, QoL-AD
Petersson and Philippou, 2016	Italy	*n* = 123	*n* = 102	CST-IT: Italian adaptation CST protocol: consisted 14 structured group sessions; Control group: treatment as usual. Twice a week, 7 weeks.	7 weeks	(−) MMSE, ↓ADAS-Cog
Zupo et al., 2025	UK	*n* = 31	*n* = 30	Intervention group: iCST App; Control group: consisted of a TAU control group and did not receive any additional interventions. For 30 min each time (e.g., two or three activities a week).	11 weeks	(−) ADAS-Cog, QoL-AD, CSDD, EQ-5D
Mancilla and Rojas, 1990	America	*n* = 23	*n* = 24	CST group: the adapted and validated version of CST for the Brazilian population (CST-Brasil); TAU: regular visits every 2/3 month to a geriatric psychiatrist and AChEI (twice a week)	7 weeks	↓Awareness of cognitive abilities
Tangney et al., 2014	Japan	*n* = 10	*n* = 10	The intervention group: CST + the inpatient rehabilitation program; The control group: the inpatient rehabilitation program. Five times a week for 8 weeks.	8 weeks	↑MMSE

RAM, Roy’s adaptation model; QOL-AD, Alzheimer’s Disease Patients’ Quality of Life Scale; CAPS, Coping and Adaptation Processing Scale; SMMSE, Standardized Mini-Mental State Examination; TAU, Treatment as usual; iCST, Individual cognitive stimulation therapy; ADAS-Cog, Alzheimer’s Disease Assessment Scale; CSDD, Cornell Scale for Depression in Dementia; CDR, The Clinical Dementia Rating; ADCS, Activities of Daily Living Inventory; HCS, The Holden Communication Scale; CAPE-BRS, Clifton Assessment Procedures for the Elderly Behavior Rating Scale; BRS, The Behavior Rating Subscale; RAID, Rating Anxiety in Dementia; QoL-AD, Quality of Life in Alzheimer’s Disease; MMSE, Mini-Mental State Examination; EQ-5D, Euro QoL five dimensions; AChEI, Cholinesterase inhibitors prescription. ↑ Indicates an increase or upregulation of the measured parameter. ↓ Indicates a decrease or downregulation of the measured parameter.

Cognitive stimulation therapy has been demonstrated to be an efficacy therapy for the treatment of cognitive dysfunction and mild to moderate dementia, with improvements in cognition, affective symptoms, behavioral challenges and quality of life demonstrated (Cao et al., 2023; Gómez-Soria et al., 2023; Wang et al., 2022; Woods et al., 2023).

As demonstrated in preceding research, CST has been shown to be an effective treatment for AD. Given the different underlying conditions of patients, it is recommended that the intervention’s activities be adapted to meet individual patient needs: (1) Assess the patient’s ability: Periodically assess the patient’s cognitive ability to adjust the difficulty of the activity. (2) Consider patients’ interests: choose activities that interest patients to increase participation and motivation. (3) Document efficacy: Regularly document the patient’s performance in cognitive activities to assess efficacy. (4) Feedback mechanisms: communicate with patients and their families to obtain feedback and make timely adjustments to the programmer. With the above implementation suggestions, we expect to be able to effectively apply cognitive stimulation therapy to provide better disease support and improved quality of life for AD patients.

## 6 Combination therapy

A generalized summary of the above individual interventions found that a single intervention can have an impact on cognitive function and quality of life in people with AD or dementia. Thus, we hypothesized that combined treatment with multiple interventions might provide additional benefit.

By searching, we found that combined lifestyle interventions were found to be more effective than single-factor interventions (Ye et al., 2023). A systematic review comparing the effectiveness of PI and NPIS to reduce symptoms of depression in people with dementia found that NPIS and multidisciplinary approaches (physical activity and CST) are highly effective interventions that benefit people with dementia (Watt et al., 2021). In addition, a systematic review showed that a healthy diet rich in fruit and vegetables and increased participation in leisure and physical activities may prevent cognitive decline and impairment in older people. Clinical trial shows that comprehensive lifestyle changes (diet and exercise) can significantly improve cognition and function after 20 weeks in many people with early dementia caused by MCI or AD (Watt et al., 2021). Trials focusing on cognitive training, improved diet and physical activity may prevent or delay cognitive decline in older adults, including those at risk of developing dementia (Castro et al., 2023).

The complexity of AD indicates that a multifaceted approach may be necessary for effective treatment. To address this, we conducted a comprehensive search for relevant reviews in the literature. One notable review has been published that summarizes the latest key advances in the treatment of AD, specifically focusing on the clinical evidence for Aβ and tau-targeted immunotherapy (Feng et al., 2026). In contrast to that review, our present study adopts a different intervention approach, providing more comprehensive evidence for NPIs. Additionally, another study explored NPIs, including light therapy, physical stimulation techniques, and acupressure, for sleep disorders in AD patients. However, this review primarily addressed a specific symptom (Montenegro et al., 2026), which limits its broader applicability. Furthermore, a separate review methodically delineates cognitive intervention therapies, such as cognitive stimulation therapy, cognitive rehabilitation, and cognitive training, emphasizing their pivotal role in non-pharmacological post-diagnosis support for dementia patients (Mulhall et al., 2026). Our review builds upon these findings by integrating a wider range of non-pharmacological strategies, thereby enhancing the understanding of comprehensive treatment options for AD.

The synergy between dietary intervention, physical exercise, and cognitive stimulation forms the cornerstone of a holistic therapeutic strategy for AD. These three modalities function as an integrated system rather than independent interventions. Dietary intervention serves as the metabolic foundation, reducing systemic inflammation and oxidative stress, which creates a favorable biological environment for neural health. Physical exercise acts as a powerful catalyst by enhancing cerebral blood flow and upregulating the expression of neurotrophic factors, such as BDNF, which are essential for synaptic maintenance. Building upon this optimized metabolic and neurotrophic base, cognitive stimulation provides the specific neural “demand” required to trigger neuroplasticity, effectively translating these physiological improvements into sustained cognitive reserve. By combining these elements, the strategy creates a “virtuous cycle”: diet stabilizes the internal environment, exercise primes the brain for adaptation, and cognitive stimulation reinforces structural integrity. This multidomain approach, consistent with the evidence-based framework established by the FINGER study (Ngandu et al., 2015), addresses the multifactorial pathology of AD more effectively than any single-domain intervention could achieve. In summary, our review not only highlights the importance of a multifaceted approach but also expands the discussion on non-pharmacological interventions, aiming to provide a more holistic perspective on the management of AD.

Above all, a combination of intervention methods has a beneficial, potentially synergistic, effect on the cognitive function of AD patients. Nevertheless, the precise mechanism of action remains to be elucidated.

## 7 Conclusion

This paper provides a concise overview of the most recent advancements in research pertaining to non-pharmacological interventions for individuals diagnosed with AD. The focus of this review is on the impact of physical activity, dietary interventions, and CST on AD patients. The findings indicate that these interventions can exert a favorable influence on cognitive impairment, enhance quality of life, reduce inflammation levels, and diminish the risk of falls.

At present, research into non-pharmacological therapies for AD remains a dynamic and evolving field. The implementation of these interventions must be considered within a pragmatic framework, as they frequently necessitate sustained patient commitment, particularly if they are expected to engender long-term effects on the patient and additional benefits for carers and family members. The objective of clinical trials is to ascertain efficacious strategies that enhance tolerability and clinical effectiveness, whilst concomitantly minimizing any potential risks associated with the intervention. Therefore, potential risks need to be controlled for various factors such as type of intervention, sample size, target population, standardized protocols, and primary and secondary outcome measures. In addition to enhancing cognitive performance, future research should concentrate on preserving autonomy in activities of daily living, enhancing patients’ quality of life, and reducing the burden on caregivers and family members.
